# Phosphatidylinositol-3 Kinase Inhibitors, Buparlisib and Alpelisib, Sensitize Estrogen Receptor-positive Breast Cancer Cells to Tamoxifen

**DOI:** 10.1038/s41598-017-10555-z

**Published:** 2017-08-29

**Authors:** I-Chun Chen, Li-Ping Hsiao, I-Wen Huang, Huei-Chieh Yu, Ling-Chun Yeh, Ching-Hung Lin, Tom Wei-Wu Chen, Ann-Lii Cheng, Yen-Shen Lu

**Affiliations:** 1 Department of Oncology, National Taiwan University Hospital, Taipei, Taiwan; 2 National Taiwan University Cancer Center, Taipei, Taiwan; 3 Graduate Institute of Oncology, National Taiwan University, Taipei, Taiwan; 4 Graduate Institute of Clinical Medicine, National Taiwan University, Taipei, Taiwan; 5 Oncology Center, National Taiwan University Hospital Hsin-Chu Branch, Hsin-Chu, Taiwan; 6 Department of Internal Medicine, National Taiwan University Hospital, Taipei, Taiwan

## Abstract

Tamoxifen is the standard first-line hormonal therapy for premenopausal women with estrogen receptor (ER)-positive metastatic breast cancer (BC). One of the crucial mechanisms underlying hormonal therapy resistance is the collateral activation of the phosphatidylinositol-3 kinase (PI3K)/AKT pathway. We explored whether PI3K inhibitors, buparlisib and alpelisib, enhance the efficacy of tamoxifen against ER-positive BC cells. We have observed a **s**ynergism between alpelisib or buparlisib and tamoxifen in the treatment for ER-positive BC cell lines harboring different PI3K alterations. Immunoblotting analysis showed alpelisib, buparlisib, or either drug in combination with tamoxifen downregulated the PI3K downstream targets in the MCF-7 and ZR75-1 cells. In the MCF-7 cells transfected with a constitutive active (myristoylated) AKT1 construct or mutant ER, the synergistic effect between alpelisib and tamoxifen was markedly attenuated, indicating that synergism depends on AKT inhibition or normally functioning ER. Combining alpelisib or buparlisib with tamoxifen also attenuated MCF-7 tumor growth in Balb/c nude mice. Our data suggest that additional PI3K blockade might be effective in enhancing the therapeutic effect of tamoxifen in ER-positive BC and support the rationale combination in clinical trials.

## Introduction

Tamoxifen is a selective estrogen receptor (ER) modulator approved for the treatment of metastatic breast cancer (MBC) patients^1, 2^ since 1977. In patients with premenopausal ER-positive MBC, tamoxifen^3^ with or without ovarian ablation by using a gonadotropin-releasing hormone agonist^4, 5^ is the major hormonal treatment. The response rate and progression-free survival of first-line tamoxifen treatment for premenopausal MBC are 30–40% and 6–7 months^6^ respectively, indicating the significance of primary or secondary resistance to tamoxifen. In contrast to the rapid advancement of aromatase inhibitors and combinations of aromatase inhibitors and targeted agents such as mammalian target of rapamycin (mTOR) inhibitor and cyclin-dependent kinase 4/6 inhibitor for the treatment of postmenopausal patients with ER-positive MBC^7–10^, the progress of hormonal therapy based treatment specifically for premenopausal ER positive-MBC has been limited over the last 20 years. Optimizing hormonal therapy based treatment is an unmet medical need for premenopausal patients with MBC.

Phosphatidylinositol-3 kinase (PI3K) is pivotal to cell proliferation^11^ and survival. Class I PI3K catalytic domains are of the following four subtypes: p110α, p110β, p110γ, and p110δ^12^. The subtypes p110α (encoded by PIK3CA) and p110β are ubiquitous across tissues, whereas p110γ and p110δ are specific to hematopoietic cells^12, 13^. PIK3CA mutation is one of the most commonly altered genes in breast cancer^6^, and its mutation rate is 40% in the ER-positive subtype^14^. Liao *et al*. has demonstrated a statistically higher PIK3CA mutation rate in premenopausal as compared to postmenopausal ER-positive BC patients^15^. Long-term exposure to tamoxifen upregulates the PI3K pathway (11), and this partially contributes to tamoxifen resistance^12, 13, 16^. Clark *et al*. has demonstrated that a PI3K inhibitor, LY294002, can potentiate tamoxifen-induced apoptosis in ER-positive cells^17^. Targeting the PI3K pathway might be a reasonable strategy to treat premenopausal ER-positive MBC^18^.

Buparlisib (BKM120) is a pan-PI3K inhibitor capable of blocking all class I PI3Ks^19^ and reducing secondary messenger production, resulting in cell growth inhibition^19, 20^. Alpelisib (BYL719) is a p110α-specific PI3K inhibitor^21^ with more favorable safety profiles than buparlisib^21^. In this study, we investigated the sensitization effect of adding PI3K inhibitors to tamoxifen to mimic the treatment of ER-positive BC in premenopausal women.

## Results

### PI3K Inhibitors Act Synergistically with Tamoxifen in Breast Cancer Cell Lines

**Figure 1:**
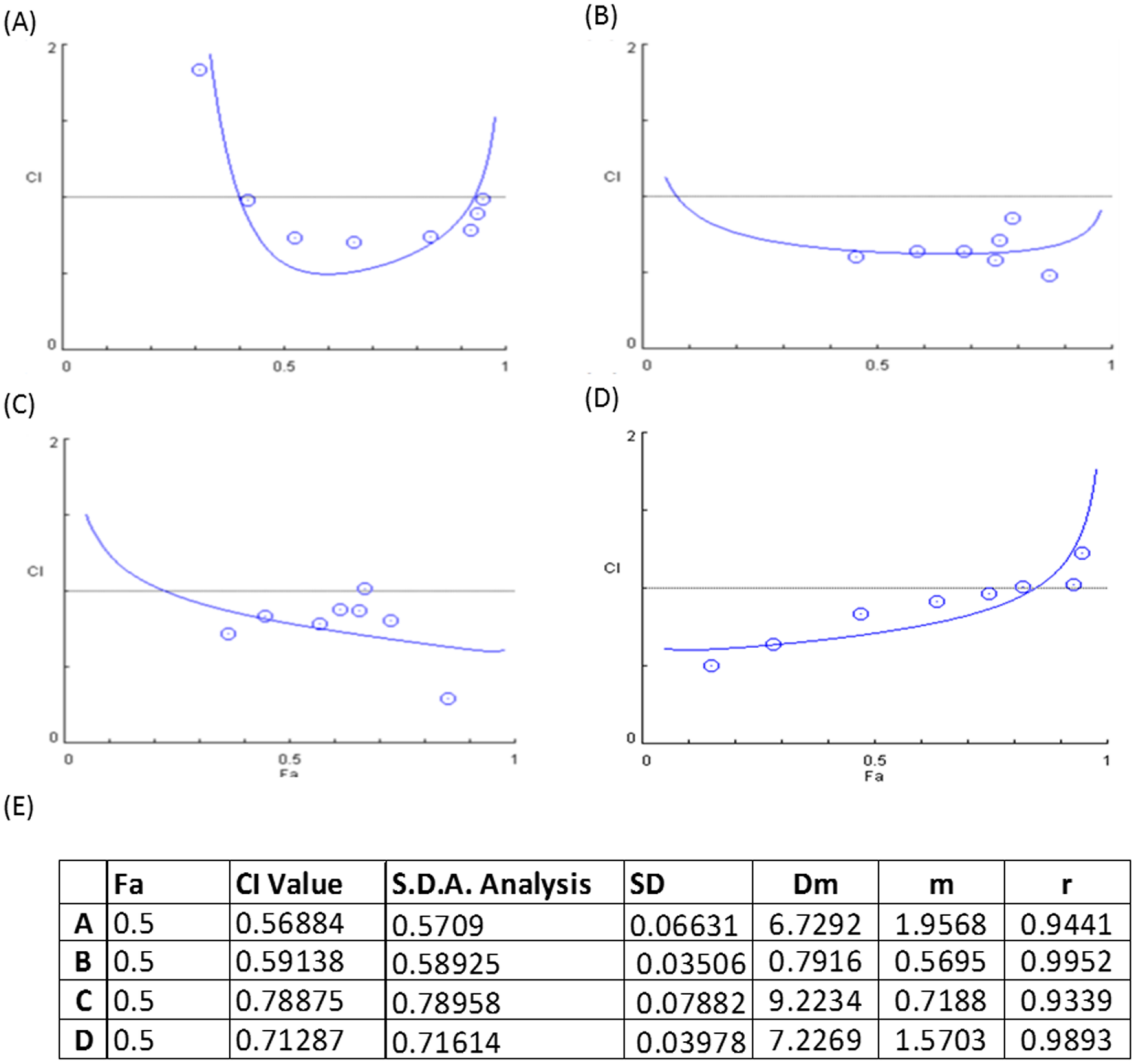
Combination Index for Tamoxifen and Alpelisib in Different ER-Positive Breast Cancer Cell Lines. (**A**) MCF-7 (PIK3CA mutation), (**B**) T47D (PIK3CA mutation), (**C**) ZR75-1 (PIK3CA wild type, PTEN loss), (**D**) HCC1500 (PIK3CA wild type, PTEN intact) and (**E**) Calculated combination index. These ER-positive breast cancer cells were treated with tamoxifen and alpelisib at different concentrations for 6 days. Combination indices were calculated to determine the relationship between drug synergism and genetic alterations in the PI3K pathway.

To investigate the efficacy of the PI3K inhibitor and its association with different mutations in the PI3K pathway, we combined tamoxifen (0–16 *μ*M) with alpelisib (0–10 *μ*M) at a constant ratio to assess the effect on breast cancer cell lines harboring different PIK3CA pathway alterations. A combination index-Fa plot is depicted to represent the median effect analysis after 6 days of treatment (Fig. 1). The combination of alpelisib and tamoxifen exhibited a synergistic effect at Fa 0.5 on both PIK3CA mutant (MCF-7 and T47D, Fig. 1A,B) and PTEN loss (ZR75-1, Fig. 1C) cell lines with a combination index <1.0 (Fig. 1E). In the HCC1500 cell line, which is a PIK3CA wild type and PTEN intact cell line, the combination index was also below 1 (Fig. 1D). This suggests that the efficacy of alpelisib and tamoxifen combination is not completely associated with specific PI3K pathway alterations.

To determine whether the synergistic effects of combining tamoxifen with specific and nonspecific PI3K inhibitors differ, we performed the median effect analysis on tamoxifen combined with a specific PI3K p110- inhibitor (alpelisib) and with a pan-PI3K inhibitor (buparlisib) in the MCF-7 cell line (Figure S1A,B). The combination indices for these two treatments at Fa 0.4 were 0.15 and 0.75, suggesting synergism. In contrast, when tamoxifen was combined with the mTOR inhibitor, everolimus, no synergism was detected in the MCF-7 cells (Figure S1C, combination index at Fa 0.4 = 1.4).

### Combination of Tamoxifen with Alpelisib or Buparlisib Alters the Phosphorylation Status of Signaling Pathways Downstream of PI3K

**Figure 2:**
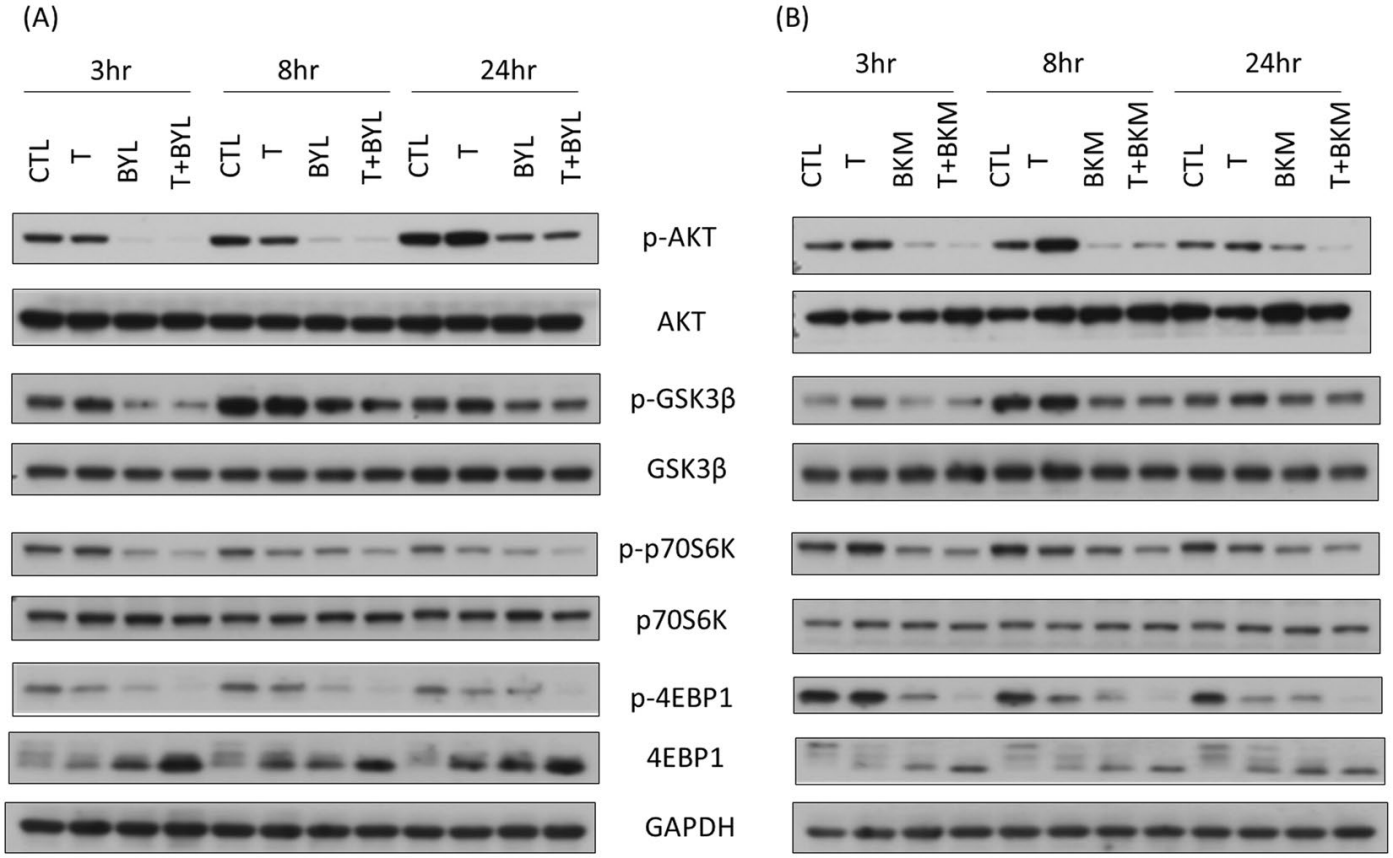
Combining Tamoxifen with (**A**) Alpelisib or (**B**) Buparlisib Attenuates Signal Transduction Downstream of PI3K in ZR75-1 Cells (tamoxifen: 8 µM; alpelisib: 5 µM; and buparlisib: 5 µM). Cells treated with a combination of tamoxifen with alpelisib or buparlisib had lower pAKT, pGSK3β, p-p70S6K, and p4EBP1 expression levels at 3, 8, and 24 hours after treatment, compared with tamoxifen-treated or control cells.

We harvested the ZR75-1 cells after a combination treatment with tamoxifen and alpelisib or tamoxifen and buparlisib at different time points to evaluate the change of different protein expressions downstream of PI3K. We observed no specific change in the total amount of the protein AKT, GSK3β, or p70S6K between the cells treated with alpelisib, tamoxifen plus alpelisib (Fig. 2A), buparlisib, or tamoxifen plus buparlisib (Fig. 2B) and the tamoxifen-treated or control cells. Tamoxifen treatment, as compared to control, resulted in increased phosphorylated AKT and GSK3B at 24 hours. As compared to tamoxifen or mock-treated cells, alpelisib, buparlisib, or a combination of either one with tamoxifen all attenuated the expression of phosphorylated AKT (pAKT), GSK3β, p70S6K, and 4EBP1 at 3, 8, and 24 hours after treatment. Compared to control or tamoxifen treatment, alpelisib alone or tamoxifen plus alpelisib (Figure S1D) reduced pAKT levels in MCF-7 cells at 3, 8, and 24 hours. Phosphorylated GSK3β (pGSK3β) and phosphor-p70S6K (p-p70S6K) to total p70S6k levels were both decreased in alpelisib alone or tamoxifen plus alpelisib treatment at 3 and 8 hours. The treatment of MCF-7 cells with buparlisib or tamoxifen plus buparlisib (Figure S1E) attenuated pAKT, p-p70S6K, and pGSK3β at 3, 8, and 24 hours after treatment. According to Bosch *et al*.^22^, ER expression increased in MCF-7 cells at 24 hours after alpelisib treatment. We also observed that the expression of ER at 24 hours was higher in the MCF-7 cells treated with alpelisib or buparlisib, with or without tamoxifen than in the control group (Figure S2A,B). The elevated ER protein expression persisted in the MCF-7 cells treated with alpelisib or tamoxifen plus alpelisib until 72 hours. However, the overexpression of ER gradually decreased in the MCF-7 cells treated with buparlisib or tamoxifen plus buparlisib after 48 hours.

### Combining Tamoxifen with PI3K Inhibitors Promotes Apoptosis

**Figure 3:**
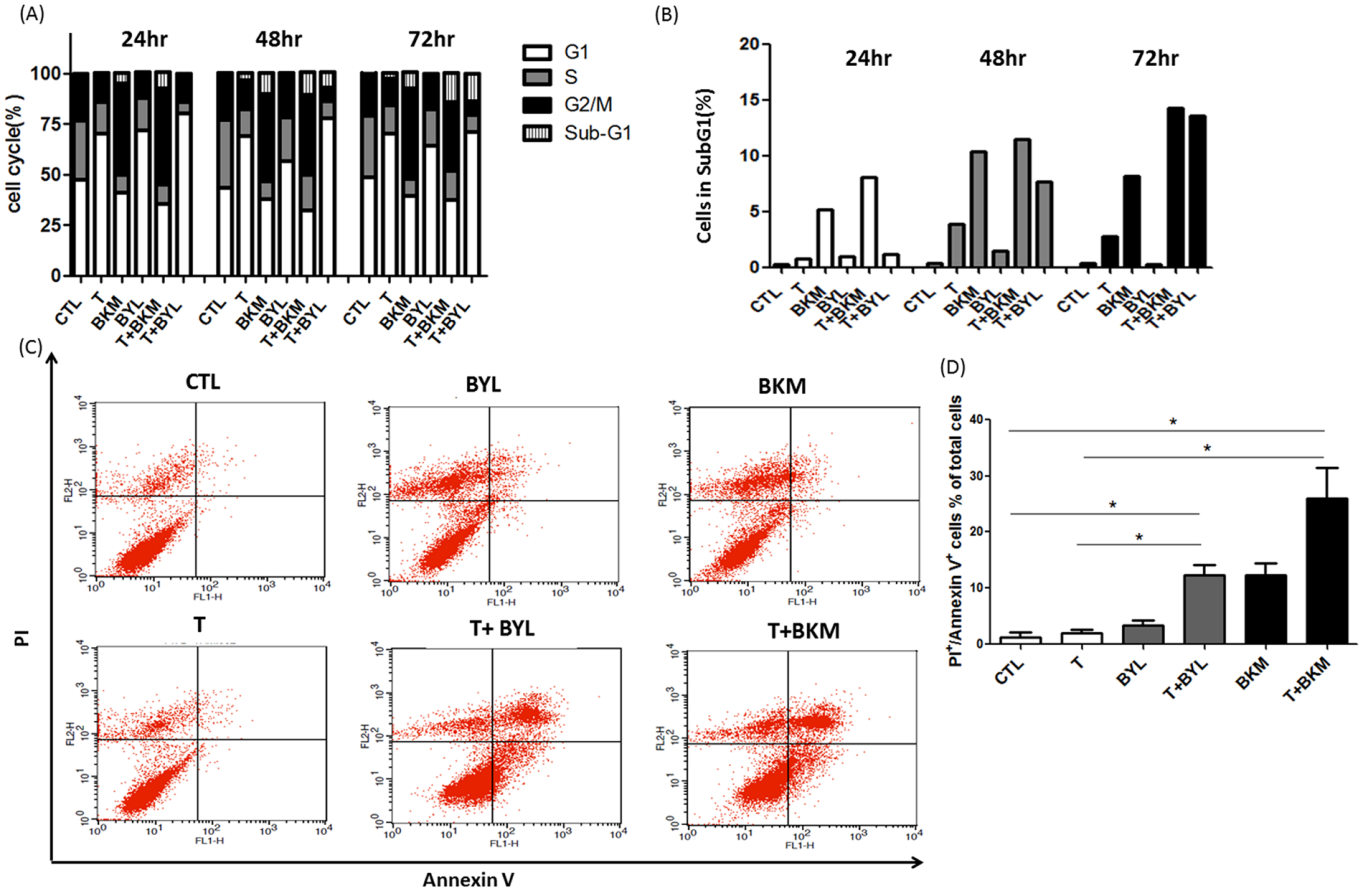
Tamoxifen Combined with Alpelisib or Buparlisib Promotes Apoptosis. (**A**) Cell cycle stage was determined through flow cytometry after the treatment of MCF-7 cells with control(CTL), tamoxifen (8 µM, T), buparlisib (5 µM, BKM), alpelisib (5 µM, BYL), tamoxifen (8 µM) + buparlisib (5 µM) (T + BKM), and tamoxifen (8 µM) + alpelisib (5 µM) (T + BYL) for 24, 48, and 72 hours. (**B**) SubG1 proportion was quantified and depicted in a bar graph. (**C**) MCF-7 cells were stained for PI and annexin V after treatment for 72 hours. Flow cytometry was used to quantify each population and the results depicted as a dot plot. (**D**) Proportions of PI and annexin V double-positive cells. (*p < 0.05) Representative figures from the three experiments are depicted in Fig. 3.

Flow cytometry was performed to determine the proportion of MCF-7 cells at different cell cycle stages after treatment. The tamoxifen plus alpelisib treatment induced G1 arrest, and the buparlisib treatment induced G2M arrest (Fig. 3A). The sub-G1 proportion in the MCF-7 cells treated with buparlisib or tamoxifen plus buparlisib increased rapidly from 24 hours after treatment, and this increase persisted after treatment for 72 hours (Fig. 3B). When the MCF-7 cells were treated with a combination of tamoxifen and alpelisib, the sub-G1 population increased after 48 hours of treatment (Fig. 3B). To understand the cell death mechanism of the tumor cells treated with tamoxifen combined with alpelisib or buparlisib, annexin V and propidium iodide stainings were performed using flow cytometry. The proportion of annexin-V-positive cells was low when the MCF-7 (Fig. 3C,D) or T47D (Figure S3A) cells were untreated or treated with tamoxifen, alpelisib, or buparlisib alone. However, the proportion of the annexin-V-positive T47D or MCF-7 cells increased significantly when the cells were treated with a combination of tamoxifen and alpelisib or buparlisib (Fig. 3C,D). The increased sub-G1 and annexin-V-positive cells are indicative of apoptotic cell death in the combination treatment.

### AKT and PIK3CA are Pivotal in the Synergistic Effect of Tamoxifen and PI3K Inhibitors

**Figure 4:**
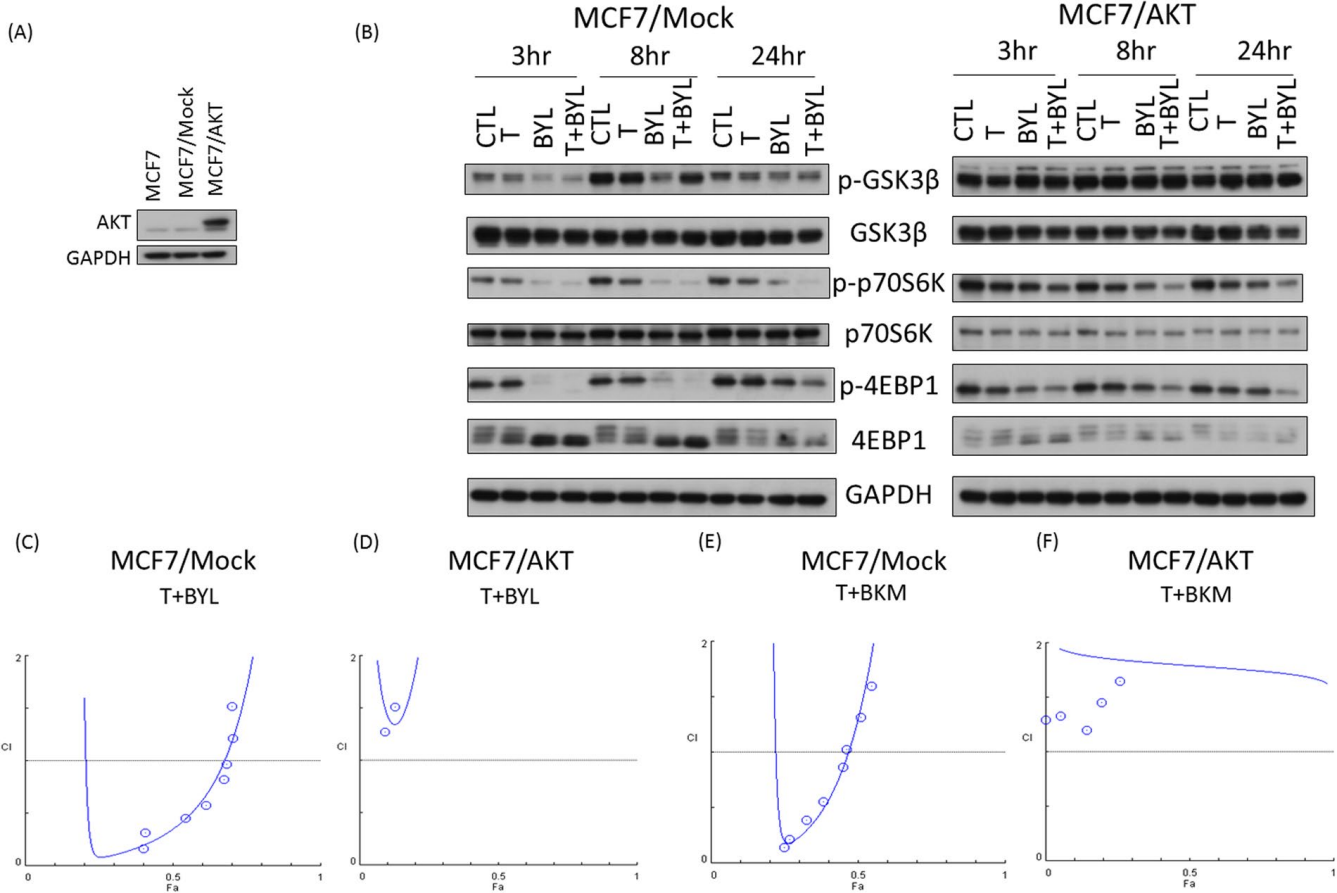
Constitutively Active AKT Abolishes the Synergistic Effect of Treatment with Tamoxifen and PI3K inhibitor. (**A**) AKT expression level was measured by Western blot analysis in MCF-7 cells without transfection (parental), cells transfected with empty vector (MCF-7/mock), and cells transfected with myristoylated AKT (MCF-7/AKT). (**B**) Comparison of p-GSK3b, pp-70S6K, and p-4EBP1 expression levels between MCF-7/mock and MCF-7/AKT cells treated with control (CTL), tamoxifen (T, 8 µM), alpelisib (BYL, 5 µM), or tamoxifen and alpelisib (T + BYL, 8 µM + 5 µM) at 3, 8, and 24 hours. Fa plots of combination indices for treatment with T + BYL in MCF-7/mock (**C**) or MCF-7/AKT (**D**) cells and treatment with T + BKM in MCF-7/mock (**E**) or MCF-7/AKT (**F**) cells.

To investigate the mechanisms underlying the synergistic effects of tamoxifen and PI3K inhibitors, T47D cells were treated with siRNAs (siRNA-control, siRNA-PIK3CA, siRNA-PIK3CB) to knockdown different isoforms of PI3K. The knock down efficiency exceeded 90% and 80% for PIK3CA and for PIK3CB, respectively (Figure S4A). The synergistic effect of tamoxifen plus alpelisib (Figure S4C, Fa 0.5, C.I. = 0.879) was attenuated when PIK3CA (Figure S4E, Fa 0.5, C.I. = 1.44), but not PIK3CB (Figure S4F, Fa 0.5, C.I. = 0.755) was inhibited by the siRNAs. PIK3CA-specific attenuation in synergism was similar when T47D cells were treated with tamoxifen plus buparlisib (Figure S4G, Fa 0.5, C.I. = 0.732) in the presence of siRNA-PIK3CA (Figure S4I, Fa 0.5, C.I. = 1.735). siRNA-PIK3CA abolished the synersitic effects of tamoxifen and PI3K inhibitors in MCF-7 cells (Figure S4K–N). These findings in T47D and MCF-7 cells suggested a PIK3CA-specific and dependent effect in tamoxifen and PI3K inhibitor synergism. To further dissect the signal transduction pathways involved in the combination treatment of tamoxifen and PI3K inhibitors, the MCF-7 cells were transfected with myristoylated-AKT to generate a constitutive active AKT expressing cell line (MCF-7/AKT, Figs 4A and S3B). MCF-7/mock cells were transfected with a vector alone. The tamoxifen plus alpelisib treatment attenuated pGSK3β, p-p70S6K, and p-4EBP1 in the MCF-7/mock cells, but this attenuation was not observed to the same extent in the MCF-7/AKT cells (Fig. 4B). In the median effect analysis, the combination indices at Fa0.4 in the MCF-7/AKT cells treated with tamoxifen plus alpelisib or tamoxifen plus buparlisib were 9.49 and 2.10 (Fig. 4D,F), indicating a loss of synergism. These results indicate that the synergistic effect of tamoxifen and alpelisib or buparlisib was lost when AKT was constitutively active, as shown in the MCF-7/AKT cells.

### Combining Tamoxifen and PI3K Inhibitor Did Not Overcome Acquired Tamoxifen Resistance or ESR1 Mutation

MCF-7/TamR is a tamoxifen-resistant cell line generated by repeated tamoxifen treatment of MCF-7 cells (25). When the MCF-7/TamR cells were treated with a combination of tamoxifen and alpelisib, no synergistic effect (Figure S3C, Fa 0.5, C.I. = 1.03, Fa 0.75, C.I. = 2.92) was observed. Also, synergism was not noted in the combination of tamoxifen with buparlisib (Figure S3D, Fa 0.5, C.I. = 0.93 and Fa 0.75, C.I. = 1.09). This absence of synergism suggests that the combination of tamoxifen and PI3K inhibitor does not overcome acquired tamoxifen resistance in MCF-7 cells.

**Figure 5:**
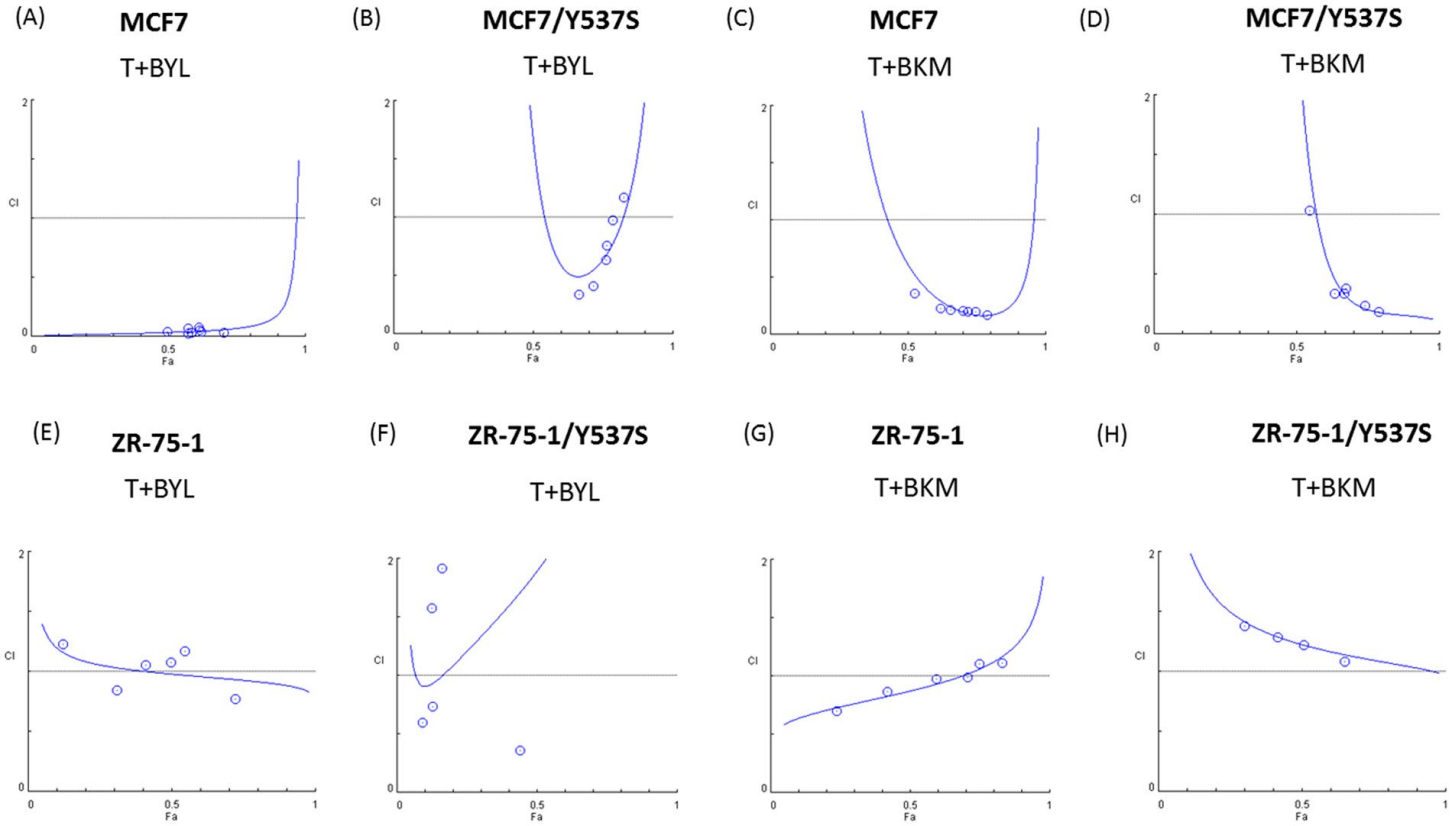
Mutant ER Results in Loss of Synergism Between Tamoxifen and PI3K Inhibitor. Y537S mutant was transfected into MCF-7 (MCF-7/Y537S) or ZR 75-1 (ZR75-1/Y537S) by lipofectamine 2000. Combination indices for T + BYL in (**A**) MCF-7, (**B**) MCF-7/Y537S, (**E**) ZR 75-1, (**F**) ZR 75-1/Y537S or tamoxifen(T) + buparisib(BKM), in (**C**) MCF-7, (**D**) MCF-7/Y537S, (**G**) ZR 75-1, (**H**) ZR 75-1/Y537S after 72 hours of treatment.

Tamoxifen can act on ER-dependent or ER-independent pathways. Tyrosine 537 is the crucial site for a functional ER^23^, and a mutation at this site confers ERα constitutive activity and acquired antiestrogen resistance^24^. We tranfected an ER mutant plasmid, pEGFP- Y537S or pEGFP-Y537C, into MCF-7 cells (MCF-7/Y537S and MCF-7/Y537C, respectively, Figure S5A–D). The synergistic effect of tamoxifen plus buparlisib (Fig. 5A, Fa 0.5, C.I. = 0.222) or tamoxifen plus alpelisib (Fig. 5C, Fa 0.5, C.I. = 0.362) was largely attenuated by the Y537S mutation (Fig. 5B, Fa 0.5, C.I. = 0.887; Fig. 5D, Fa 0.5, C.I. = 0.825) and Y537C mutation. We transfected a Y537S mutant into ZR75-1 cell line (ZR-75-1/Y537S, Figure S5E,F). While testing the combination effects, the presence of Y537S in ZR75-1 cell resulted in attenuated synergism in both tamoxifen plus alpelisib (Fig. 5E,F, Fa 0.5, C.I. = 0.979 and 1.894) and tamoxifen plus buparlisib (Fig. 5G,H, Fa 0.5, C.I. = 0.872 and 1.224) treatments. The results obtained from both MCF-7/Y537S and ZR75-1/Y537S cells indicated that the synergism relies on ER with ligand-dependent activation.

### Synergistic Effect of Tamoxifen and PI3K Inhibitors *in vivo*

**Figure 6:**
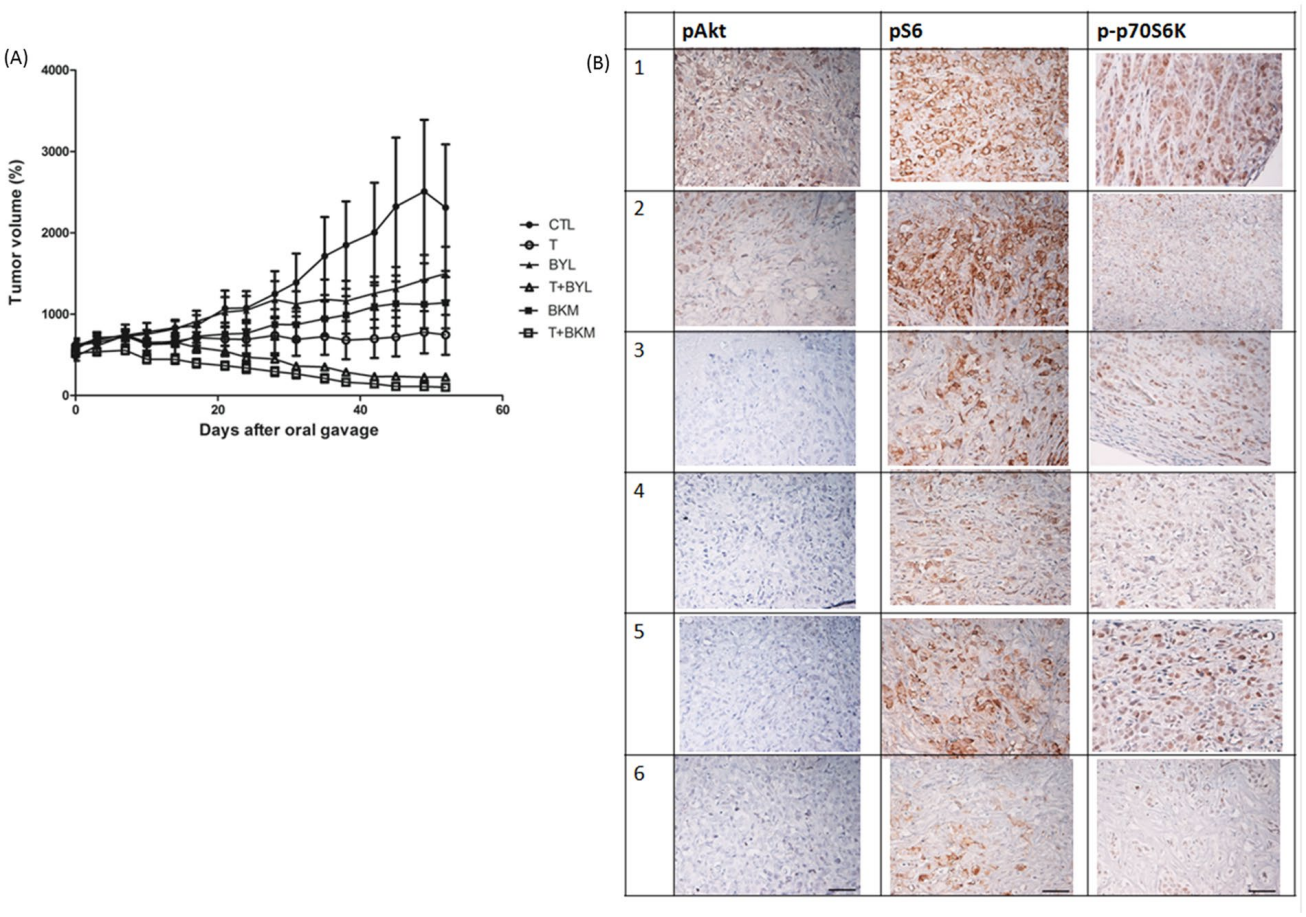
Treatment with Tamoxifen and a PI3K Inhibitor Controls MCF-7 Tumor Growth *In Vivo*. Female Balb/c nude mice were inoculated with MCF-7 cells at the flank and randomized into six different treatment groups when the tumor volume reached 500 mm^3^. (**A**) Tumor volumes measured using a caliper for treatments with CTL (solid circles; PBS), T (open circles; tamoxifen, 50 mg/kg), BYL (solid triangles; alpelisib, 35 mg/kg), T + BYL (open triangles; tamoxifen + alpelisib, 50 mg/kg + 35 mg/kg), BKM (solid squares; 25 mg/kg), and T + BKM (open squares; tamoxifen + buparlisib, 50 mg/kg + 25 mg/kg). The figure is representative of two independent experiments (N = 5 and 7 in each treatment group). (**B**) Representative figures of paraffin-embedded MCF-7 tumors from (1) CTL, (2) T, (3) BYL, (4) T + BYL, (5) BKM, and (6) T + BKM treatments were stained for pAKT, pS6, and p-p70S6K. All images were obtained using a 10x eyepiece and 40x objective lens (scale bar = 50 µm).

The antitumor effects of tamoxifen, alpelisib, and buparlisib, as well as combinations of tamoxifen and alpelisib or tamoxifen and buparlisib, were tested in an *in vivo* xenograft model. The treatments were started after tumors were more than 500 mm^3^. When the mice were treated with tamoxifen alone, the tumor became stabilized, with size similar to that before treatment. The tumor size of those treated with alpelisib or buparlisib alone was larger than that of those treated with tamoxifen alone (Fig. 6A). When tamoxifen was combined with alpelisib or buparlisib, the average tumor size was less than 200 mm^3^ on the day of sacrifice. The tumor size differences between control and tamoxifen (p = 0.0022), alpelisib plus tamoxifen (p = 0.0009), or buparlisib plus tamoxifen (p = 0.0007) were statistically significant. The tumor sizes observed for the combination treatments were all significantly smaller than those observed for the single-agent treatments (p < 0.005, Table S1). The result suggests that tamoxifen alone might suppress MCF-7 growth in mice, but a combination of tamoxifen and PI3K inhibitor can lead to tumor shrinkage.

The mice were sacrificed after 7 weeks of treatment. IHC stainings for phosphorylated AKT (pAKT), phosphorylated S6 (pS6), and phosphorylated p70S6 were performed on tumor-derived samples. The pAKT, pS6, and p-p70S6 expression levels in the tamoxifen plus alpelisib (Fig. 6B–4) or tamoxifen plus buparlisib (Fig. 6B–6) groups were attenuated as compared with those in the tamoxifen (Fig. 6B–2) or control (Fig. 6B–1) group. These results are consistent with the *in vitro* results.

## Discussion

Our study reveals the synergistic effect of combining PI3K inhibitors with tamoxifen, as indicated by a median effect analysis *in vitro* and by remarkable tumor shrinkage in an MCF-7 xenograft model. This synergism depends on the presence of PIK3CA, AKT, and wild type ER.

In our study, the synergistic effect of tamoxifen and PI3K inhibitors were not completely related to PIK3CA status. The synergism was more prominent in PIK3CA mutant cell lines (MCF-7 and T47D) than in PIK3CA wild type cell lines (ZR75-1, HCC1500). The synergism of tamoxifen plus PI3K inhibitors in wild type ZR75-1 cell line is consistent with the findings of a biomarker study from the BOLERO-2 trial^25–27^ and a preclinical cell model presented by Fritsch *et al*.^21^. In 227 patients with available tumors for mutational analysis, the BOLERO-2 trial observed that PIK3CA mutation status was not predictive of the efficacy of everolimus^25–27^. In addition, preclinical cell models presented by Fritsch *et al*. revealed that PIK3CA mutation caused increased alpelisib sensitivity, but PIK3CA wild type cell lines were still selectively sensitive to alpelisib^21^. PIK3CA mutation in circulating tumor DNA, but not primary tumor is associated with longer progression free survival (PFS) in BELLE2 study^28^. Di Leo *et al*. has reported that PIK3CA mutation in both tumor and circulating tumor DNA is associated with higher PFS in BELLE 3 study^29^. Their findings might help to explain the lower combination index in PIK3CA mutant than wild type BC cell lines in our study.

The synergistic effect of tamoxifen plus buparlisib or alpelisib was abolished when siRNA-PIK3CA was used to knockdown PIK3CA in T47D and MCF-7 cells. This indicated a PIK3CA-specific and -dependent synergistic effect between tamoxifen and PI3K inhibitors. The signaling pathways downstream of PI3K were altered by the combination of tamoxifen and buparlisib or alpelisib. The expression of p-AKT, p-GSK3b, p-p70S6K, and p-4EBP1 was all reduced by the combination of tamoxifen with either buparlisib or alpelisib in ZR75-1 and MCF-7 cells. Although the signals downstream of PI3K in ZR 75-1 and MCF-7 cells were attenuated at different time points, the patterns observed in these two cell lines were similar. This finding is consistent with Clark’s study^17^ that they have shown an association of AKT expression in MCF7 cells when treated with Tamoxifen plus LY294002. In our study, when myristoylated-AKT was transfected into the MCF-7 cells, the synergistic effect was abolished. Our result, as compared to Clark’s study, further indicates that the synergistic effect is mediated through the suppression of p-AKT. In addition, when an activated mutant ER was transfected into the MCF-7 or ZR 75-1 cells, the synergistic effect disappeared. These results suggest that the AKT and non-constitutively active ER are mandatory for achieving the synergistic effect of tamoxifen and alpelisib or tamoxifen and buparlisib. Bosch *et al*.^30^ discovered that increased ER expression might be an adaptive mechanism for cells treated with PI3K inhibitors. Our findings regarding the elevated ER expression levels after PI3K inhibitor treatment also recapitulate the phenomenon observed by Bosch *et al*. However, we also noted that when the MCF-7 cells were treated with buparlisib or tamoxifen plus buparlisib, the ER level declined after 48 hours. These findings indicate a potential difference between the effects of combining tamoxifen with PI3K alpha-specific inhibitors and with PI3K general inhibitors in a clinical setting, and this phenomenon therefore warrant further investigation. According to the results of Bosch *et al*. and those of our study, we might speculate that the ER and PI3K/AKT pathways function similar to a bidirectional circuit in which each pathway serves as a backup mechanism for the other pathway. Therefore, targeting ER and PI3K pathways together might not only produce a synergist effect but also delay the occurrence of resistance to either treatment alone.

In our study, the antitumor effect engendered by treatment with tamoxifen plus buparlisib was not different from that engendered by treatment with tamoxifen plus alpelisib in the MCF-7 xenograft model. Costa *et al*.^31^ measured PIP3 levels in cancer cells treated with alpelisib and determined that p110β evolved as an escape mechanism for p110α-specific inhibitors. Alpelisib only resulted in attenuated tumor growth in BT474 xenograft model. Tumor regression is noted when they combined p110α specific (alpelisib) and p110β specific inhibitors (KIN-193), This might explain why treatment with tamoxifen plus buparlisib induced slightly more tumor regression than did treatment with tamoxifen plus alpelisib in our MCF-7 xenograft model. However, a drug with broader spectrum of targets inhibition is usually associated with higher clinical toxicity. Therefore, the better clinical choice between buparlisib and alpelisib awaits the results from current clinical trials.

Some questions remain unanswered in our study. The combination index for tamoxifen and alpelisib or tamoxifen and buparlisib did not show synergism in the MCF-7/TamR cells, which have acquired tamoxifen resistance. Although mechanisms underline this result necessitates further investigation, it supports conventional trial design of enrolling only MBC patients without prior tamoxifen therapy for metastatic disease to receive the combination of tamoxifen and targeted agent (such as B-YOND trial, NCT02058381). For more than 20 years, tamoxifen with or without ovarian ablation has been the only Food and Drug Administration-approved hormonal therapy for premenopausal MBC patients. Our findings regarding the synergistic effects of tamoxifen plus PI3K inhibitors potentially offered another optimized treatment option for premenopausal MBC patients.

## Material and Methods

### Cell Lines and Reagents

T47D (PIK3CA mutant), HCC1500 (PIK3CA wild type and PTEN intact), and ZR75-1 (PIK3CA wild type and PTEN loss) cell lines were obtained from Dr. Dan R. Robinson’s laboratory. (University of Michigan Medical School, Ann Arbor, Michigan,United States). The MCF-7 (PIK3CA mutant) cell line was a kind gift from Dr. Jose Baselga’s laboratory (Memorial Sloan Kettering Cancer Center, New York, NY). We did not further test or authenticate the cells. The cells were cultured according to the manufacturer’s manual. Annexin V and propidium iodide (PI) were purchased from Cell Signaling Technology (Danvers, MA, United States). Moreover, 4-hydroxy-tamoxifen (tamoxifen) was purchased from Sigma Aldrich (St. Louis, MO, United States). Buparlisib and alpelisib compounds for *in vitro* and *in vivo* studies were supplied by Novartis (Basel, Switzerland). Thiazolyl blue tetrazolium bromide (MTT) was purchased from Gold Biotechnology (St Louis, MO, United States). siRNAs (siRNA-control, siRNA-PIK3CA, siRNA-PIK3CB) were purchased from GE Dharmacon (Lafayette, CO, United States) and used according to manufacturer’s instructions.

### Cell Viability Assay and Median Effect analysis

The cells were treated with investigational compounds in 96-well flat-bottomed plates (Corning, Inc.) for 6 days, with replenishment of the medium and investigational compounds every 3 days. Cell viability was examined using a previously described MTT method^32^. Median effect analysis^33^ was performed to evaluate the combination effect of tamoxifen and alpelisib or tamoxifen and buparlisib. Briefly, we plotted the dose-survival curve for tamoxifen, alpelisib, and buparlisib as a single agent across a range of different concentrations and in different cell lines. Subsequently, all of the compounds were tested at a constant 1:1 or 1:10 ratio where stated with fraction affected (Fa) ranging from 0.1 to 0.9. Combination index (C.I.) and Fa plot was depicted to represent the combination effect.

### Cell Cycle and Apoptosis Detection

Flow cytometry was performed to determine the proportion of cells at different stages of the cell cycle and the proportion of the sub-G1 population after various treatments. The extent of apoptosis was also analyzed using an Annexin-V FITC apoptosis kit (Invitrogen, CA). The cells were stained with FITC-conjugated Annexin-V and propidium iodide, according to the manufacturer’s instructions, and analyzed through flow cytometry using a FACScan (BD Biosciences, CA). Data analysis was conducted using CELLQuest (BD Biosciences, CA).

### Western Blot Analysis

Following the different treatments, as indicated in the figure legends, the cells were lysed, and the protein concentration of each lysate was determined. Subsequently, aliquots (15 μg) of the lysates were subjected to western blot analysis. The following antibodies were used: anti-phospho-AKT (Ser473), anti-S6, anti-phospho-S6 (Ser235/236), anti-GSK3B, anti-phospho-GSK3B (Ser9), anti-phospho-70S6K, anti-phospho-p70S6K (Thr421/Ser424), anti-4EBP1, anti-phospho-4EBP1 (Ser65), anti-PI3K-p110α, and anti-PI3K-p110β from Cell Signaling Technology (Danvers, MA, United States), and anti-AKT from Santa Cruz Biotechnology (Dallas, TX, United States). All of the dilution and preparation of antibodies were performed according to manufacturer’s datasheet.

### Transfection and Stable Clone

Myristoylated AKT1 plasmid was purchased from Millipore (Billerica, MA, United States). In addition, p-EGFP-ERY537S and p-EGFP-ERY537C mutant plasmids were purchased from GenScript (Piscataway, NJ, United States). All of these plasmids were transfected into the MCF-7 or ZR 75-1 cells by using a lipofectamine 2000 kit (Life Technologies, Grand Island, NY, United States) with G418 selection. Successful mutant transfection was confirmed through DNA sequencing (ABI 3730, Foster City, CA, United States) at the Genomic Core Lab in our institution and through GFP detection under a fluorescence microscopy (Leica, Wetzlar, Germany) in the laboratory.

### Xenograft Model

All mouse studies were conducted using animal protocols approved by the Institutional Animal Care and Use Committee of the National Taiwan University Medical College in accordance with institutional guidelines. An estrogen pellet (0.72 mg 90-day release, Innovative Research of America, Sarasota, Florida) was implanted on 5-week-old female BALB/cAnN.Cg-Foxn1nu/CrlNarl nude mice (National Laboratory Animal Center, Taipei, Taiwan) 2 days before 2 × 10^6^ MCF-7 cell inoculation. When the average tumor size reached 500 mm^3^, the mice were randomized according to their tumor size into six treatment groups: phosphate-buffered saline (PBS; control), tamoxifen (50 mg/kg), alpelisib (35 mg/kg), buparlisib (25 mg/kg), alpelisib (35 mg/kg) + tamoxifen (50 mg/kg), and buparlisib (25 mg/kg) + tamoxifen (50 mg/kg). These treatments were administered by gavage feeding once daily for 6 days per week. The tumor size was measured weekly using a caliper and calculated as 0.5 × longest length × (width)^2^. The mice were sacrificed after 7 weeks of treatment, and the tumors were harvested for immunohistochemical (IHC) staining. IHC images were acquired using a Leica DMR microscope (Heidelberg, Germany) at our institution.

## Electronic supplementary material

Supplementary information
