# Supplementary material for: Phosphatidylinositol-3 Kinase Inhibitors, Buparlisib and Alpelisib, Sensitize Estrogen Receptor-positive Breast Cancer Cells to Tamoxifen
Source: Sci Rep. 2017 Aug 29;7:9842. doi: 10.1038/s41598-017-10555-z (PMC5574981; doi:10.1038/s41598-017-10555-z)
Supplement: Supplementary file 1 — Supplementary information [file 41598_2017_10555_MOESM1_ESM.pdf]

**Supplemental Information**

# Phosphatidylinositol-3 Kinase Inhibitors, Buparlisib and Alpelisib, Sensitize Estrogen Receptor-positive Breast Cancer Cells to Tamoxifen

I-Chun Chen, Li-Ping Hsiao, I-Wen Huang, Huei-Chieh Yu, Ling-Chun Yeh, Ching-Hung Lin, Tom Wei-Wu Chen, Ann-Lii Cheng, and Yen-Shen Lu

Supplemental Figure 1: Median Effect Analysis and western blotting results in MCF-7 cells

MCF-7 cells were treated with (A) T + BYL, (B) T + BKM, and (C) T + everolimus for 72 hours. Immunoblotting was performed at 3, 8, and 24 hours after control (CTL), tamoxifen (T), alpelisib (BYL), and T+BYL in (D) or buparlisib (BKM) and T+BKM in (E).

Supplemental Figure 2:

ER expression level in MCF-7 cells treated with (A) CTL, T, BYL, T + BYL or (B) CTL, T, BKM, and T + BKM at 3, 8, 24, 48, and 72 hours.

Supplemental Figure 3:

(A) Proportion of apoptotic cells in T47D cells treated with CTL, T, BYL, T+BYL, BKM, and T + BKM for 72 hours. (B) MCF-7 cells transfected with myristolyated AKT. Clones 5 and 8 were chosen for subsequent experiments. Median effect analysis for tamoxifen-resistant MCF-7 (MCF-7/TamR) treated with T + BYL (C) or T + BKM (D) for 72 hours.

Supplemental Figure 4:

(A) Knockdown efficiency of PI3K-p110 $\alpha$ and PI3K-p110 $\beta$ by different siRNAs, (1) wild type (2) siRNA-control (3) siRNA-PIK3CA and (4) siRNA-PIK3CB, in T47D cells. (B) Knockdown efficiency of PI3K-p110 $\alpha$ by (1) wild type (2) siRNA-control (3) siRNA-PIK3CA, in MCF7 cells. Median effect analysis for T+BYL in T47D cells: (C) wild type (D) siRNA-control (E) siRNA-PIK3CA (F) siRNA-PIK3CB or in MCF-7 cells: (K) wild type (L) siRNA-PIK3CA. Median effect analysis for T+BKM in T47D cells: (G) wild type (H) siRNA-control (I) siRNA-PIK3CA (J) siRNA-PIK3CB or in MCF-7 cells: (M) wild type (N) siRNA-PIK3CA. All

of the siRNA were given at 200pM according to manufacturer's manual. Both the western blotting and median effect analysis were performed after 72 hours of treatment.

Supplemental Figure 5: MTT Assay of Alpelisib or Buparlisib in Different ER Mutant MCF-7 Cells

MCF-7 cells transfected with Y537S (MCF-7/Y537S) or Y537C (MCF-7/Y537C) mutant plasmids were treated with (A) alpelisib or (B) buparlisib.

DNA sequencing analysis to confirm the presence of the mutant plasmid (TAT to AGT) in MCF7 cells (C) or ZR 75-1 cells (E). Detection of GFP under fluorescence microscopy (Leica, 200x) in MCF-7 cells (D) or ZR 75-1 cells (F)

Supplemental Figure 6: Uncropped western blot figures for Figure 2A and 2B

Uncropped original western blot figures for Figure 2A (A-C) and Figure 2B (D-F)

Supplemental Figure 7: Uncropped western blot figures for Figure 4B

The western blots that were cropped into Figure 4B were marked in blue square.

Supplemental Figure 8: Uncropped western blot figures for Supplemental Figure 1D, 1E, 2A, and 2B

Uncropped original western blot figures for Supplemental Figure 1D (A), Supplemental Figure 1E (B), Supplemental Figure 2A (C), and Supplemental Figure 2B (D)

Supplemental Figure 9: Uncropped western blot figures for Supplemental Figure 3B, 4A, and 4B

Uncropped original western blot figures for Supplemental Figure 3B (A), Supplemental Figure 4A (B), and Supplemental Figure 4B (C)

Supplement Table 1: Tumor Size Comparison Between Treatment Groups

Unpaired t-test was used to compare treatment effects between groups. p-value is listed in the table.

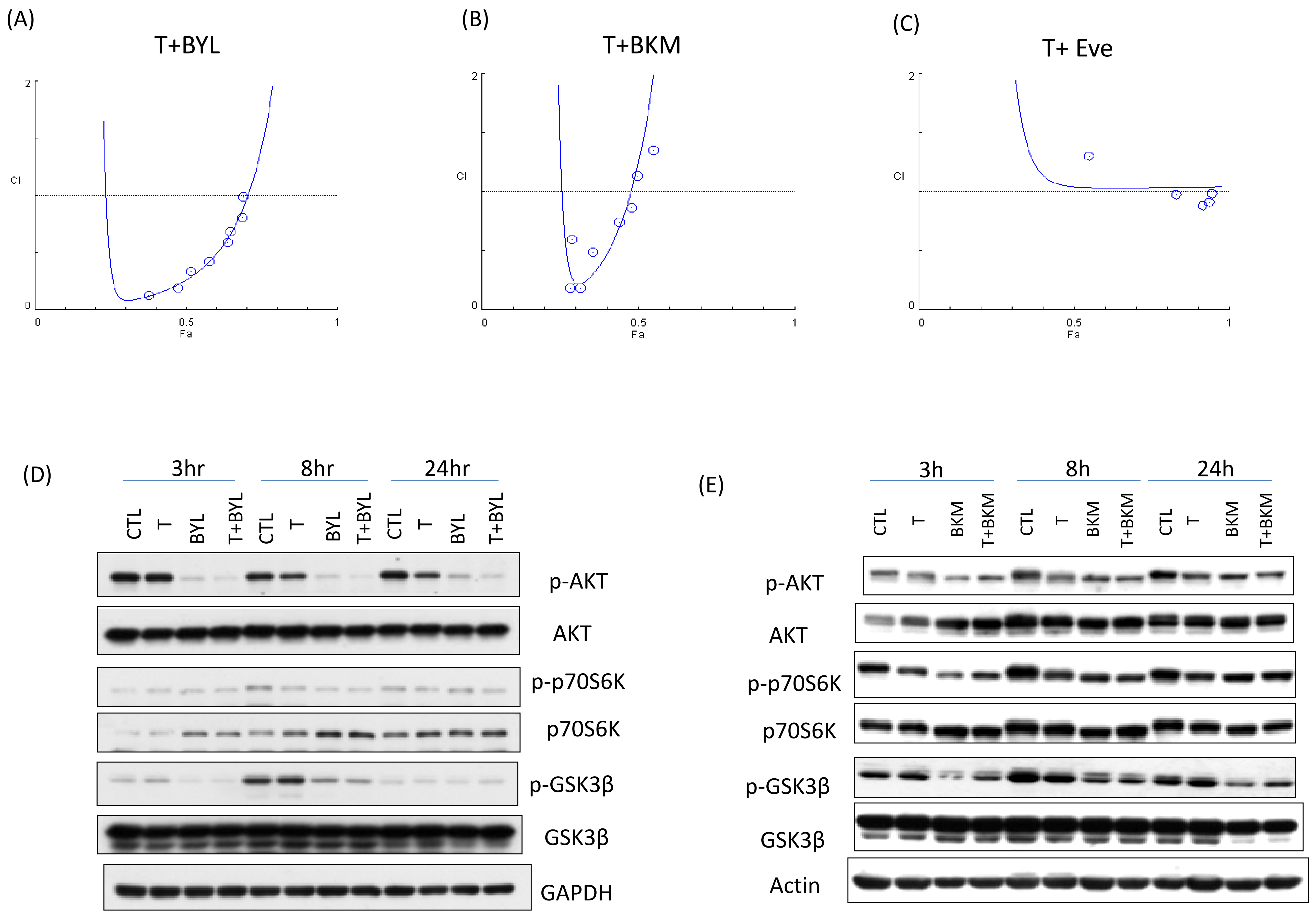

(A)
T+BYL
CI
Fa
(B)
T+BKM
CI
Fa
(C)
T+ Eve
CI
Fa
(D)
3hr
8hr
24hr
CTL
T
BYL
T+BYL
p-AKT
AKT
p-p70S6K
p70S6K
p-GSK3β
GSK3β
GAPDH
(E)
3h
8h
24h
CTL
T
BKM
T+BKM
p-AKT
AKT
p-p70S6K
p70S6K
p-GSK3β
GSK3β
Actin

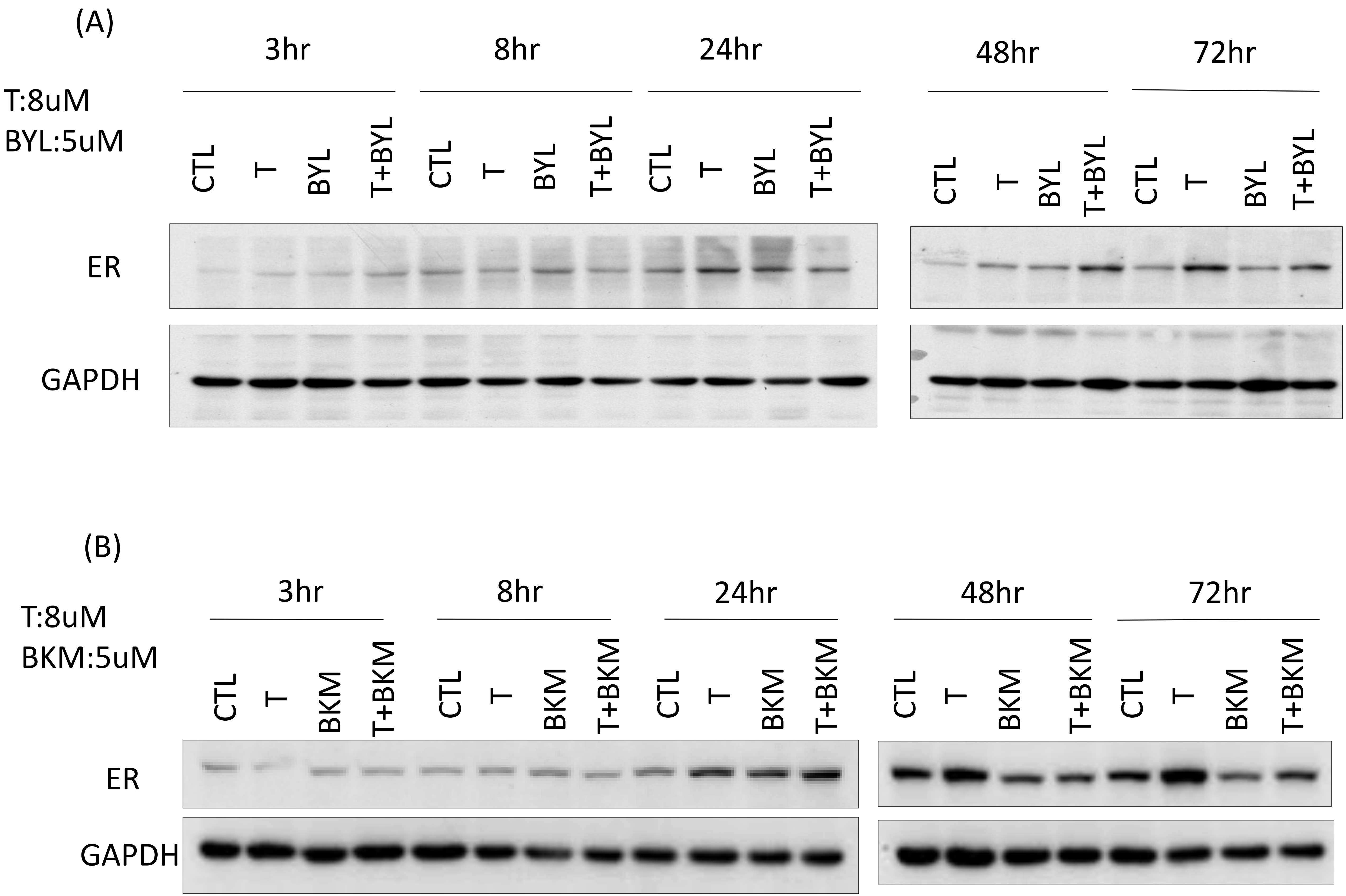

(A)
T:8uM
BYL:5uM
3hr
8hr
24hr
48hr
72hr
CTL
T
BYL
T+BYL
ER
GAPDH
(B)
T:8uM
BKM:5uM
3hr
8hr
24hr
48hr
72hr
CTL
T
BKM
T+BKM
ER
GAPDH

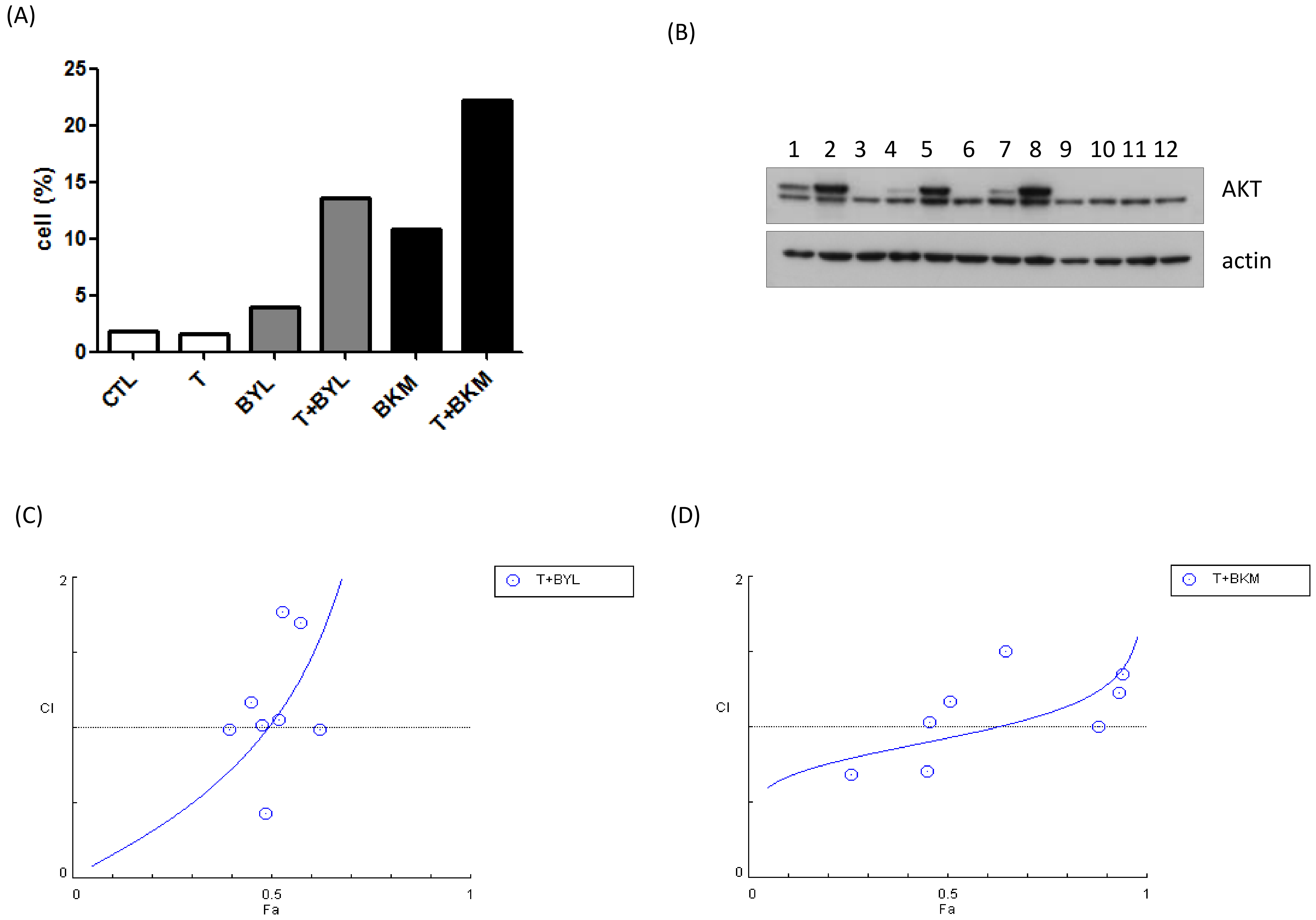

(A)
cell (%)
25
20
15
10
5
0
CTL
T
BYL
T+BYL
BKM
T+BKM
(B)
1 2 3 4 5 6 7 8 9 10 11 12
AKT
actin
(C)
T+BYL
2
CI
0
0
0.5
1
Fa
(D)
T+BKM
2
CI
0
0
0.5
1
Fa

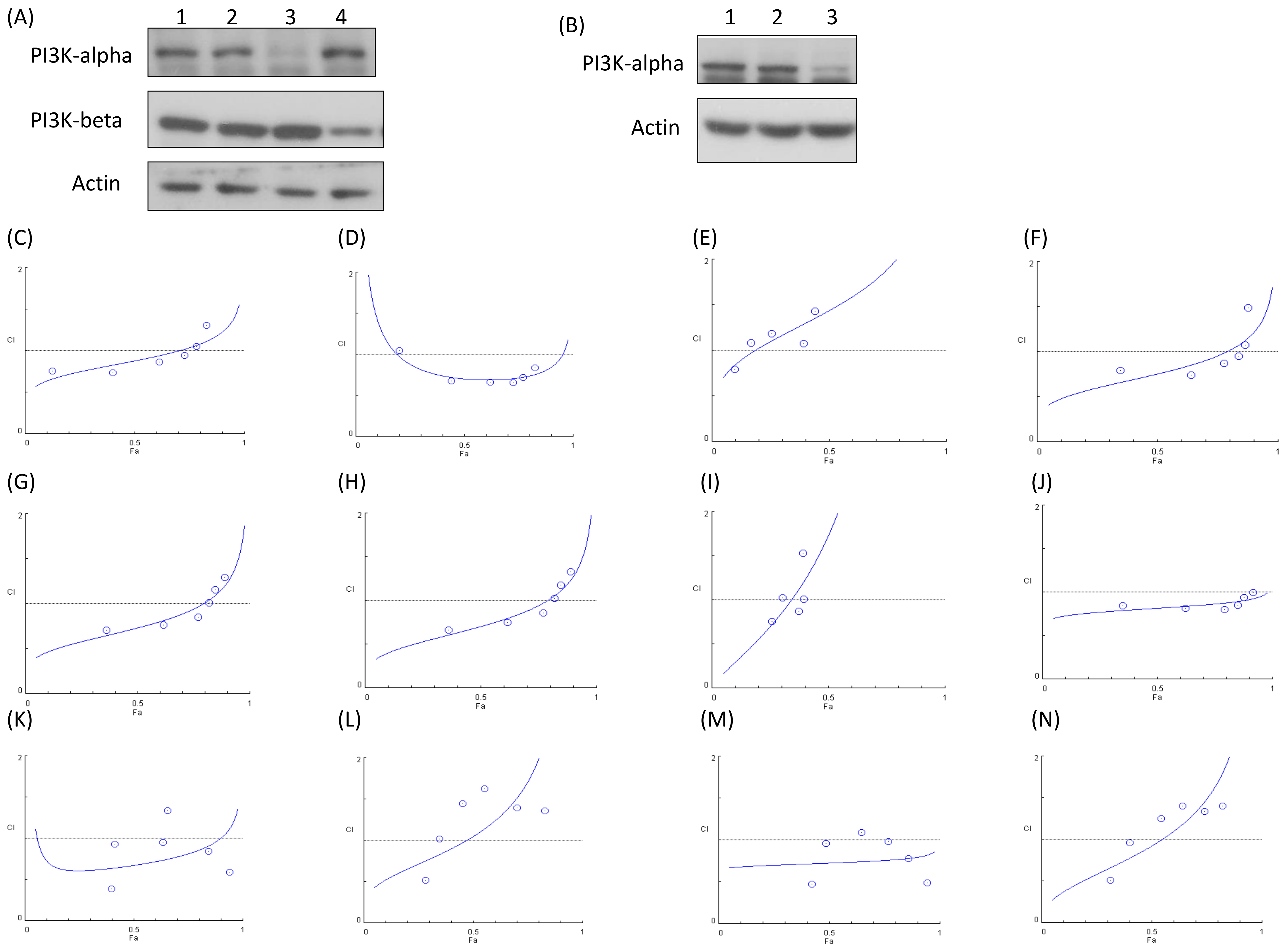
(A)
1 2 3 4
PI3K-alpha
PI3K-beta
Actin
(B)
1 2 3
PI3K-alpha
Actin
(C)
(D)
(E)
(F)
(G)
(H)
(I)
(J)
(K)
(L)
(M)
(N)
CI
Fa
0
0.5
1
2

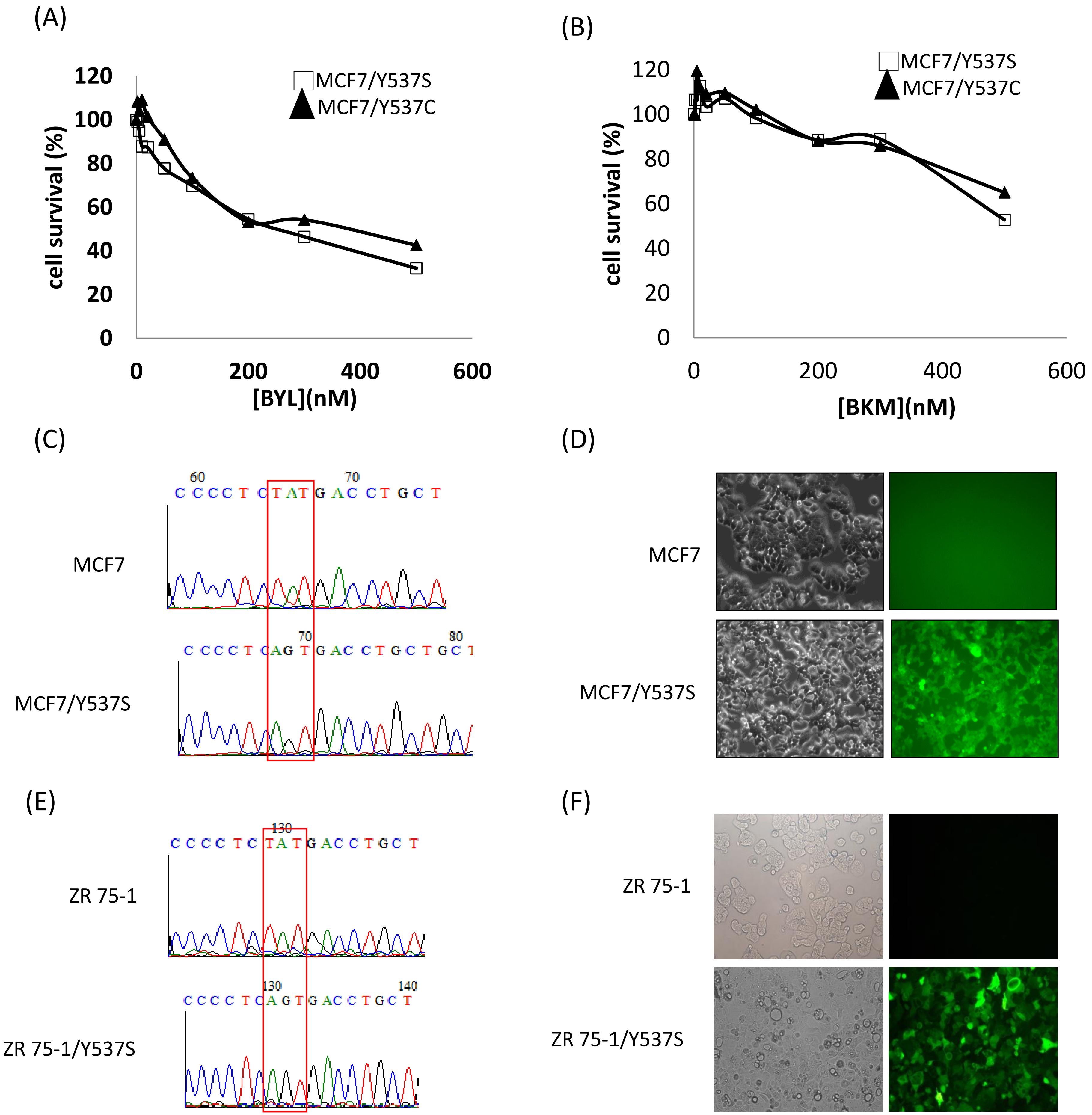
(A)
120
100
80
60
40
20
0
cell survival (%)
0
200
400
600
[BYL](nM)
MCF7/Y537S
MCF7/Y537C
(B)
120
100
80
60
40
20
0
cell survival (%)
0
200
400
600
[BKM](nM)
MCF7/Y537S
MCF7/Y537C
(C)
MCF7
60
70
C C C C T C T A T G A C C T G C T
MCF7/Y537S
70
80
C C C C T C A G T G A C C T G C T G C T
(D)
MCF7
MCF7/Y537S
(E)
ZR 75-1
130
C C C C T C T A T G A C C T G C T
ZR 75-1/Y537S
130
140
C C C C T C A G T G A C C T G C T
(F)
ZR 75-1
ZR 75-1/Y537S

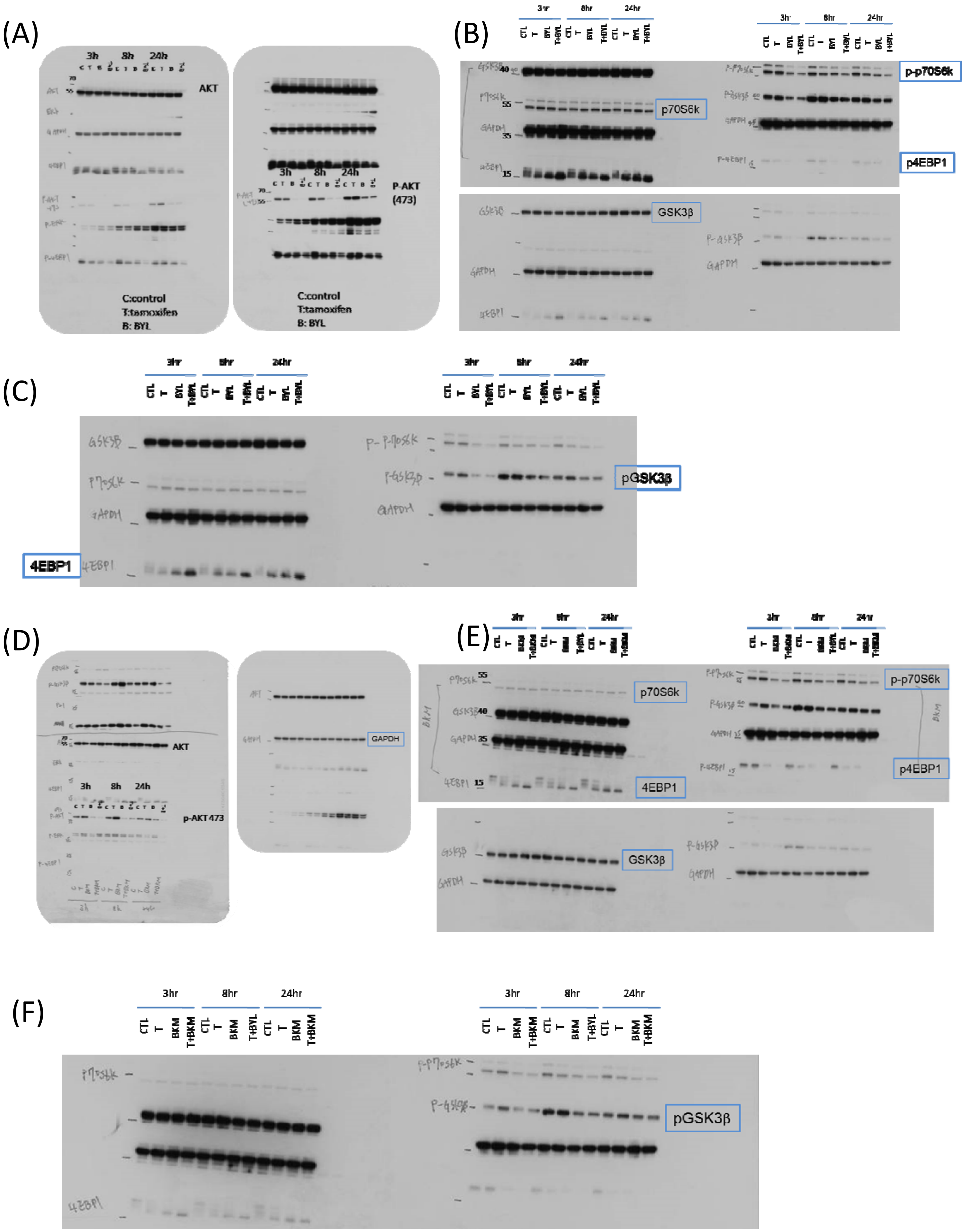
(A)
3h 8h 24h
AKT
P-AKT (473)
C:control
T:tamoxifen
B: BYL
(B)
3hr 8hr 24hr
CTL T BYL T+BYL
p-p70S6k
p70S6k
p4EBP1
GSK3β
(C)
3hr 8hr 24hr
CTL T BYL T+BYL
4EBP1
pGSK3β
(D)
AKT
3h 8h 24h
p-AKT 473
GAPDH
(E)
3hr 8hr 24hr
CTL T BKM T+BKM
p70S6k
p-p70S6k
p4EBP1
4EBP1
GSK3β
(F)
3hr 8hr 24hr
CTL T BKM T+BKM
T+BYL
pGSK3β

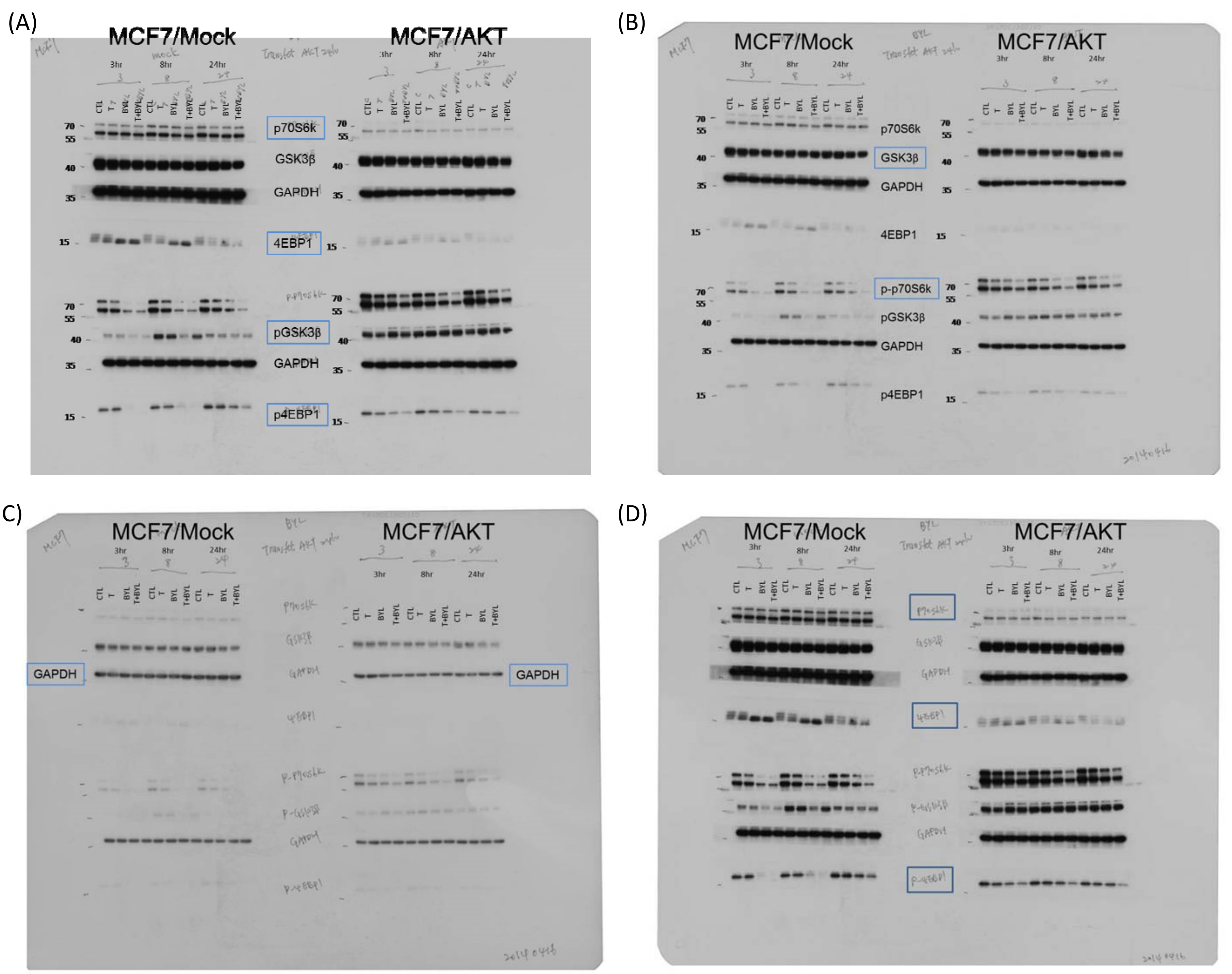
(A)
MCF7/Mock
MCF7/AKT
3hr 8hr 24hr
CTL T BYL T+BYL
p70S6k
GSK3β
GAPDH
4EBP1
pGSK3β
GAPDH
p4EBP1
70 55 40 35 15
(B)
MCF7/Mock
MCF7/AKT
3hr 8hr 24hr
CTL T BYL T+BYL
p70S6k
GSK3β
GAPDH
4EBP1
p-p70S6k
pGSK3β
GAPDH
p4EBP1
70 55 40 35 15
(C)
MCF7/Mock
MCF7/AKT
3hr 8hr 24hr
CTL T BYL T+BYL
GAPDH
GAPDH
(D)
MCF7/Mock
MCF7/AKT
3hr 8hr 24hr
CTL T BYL T+BYL

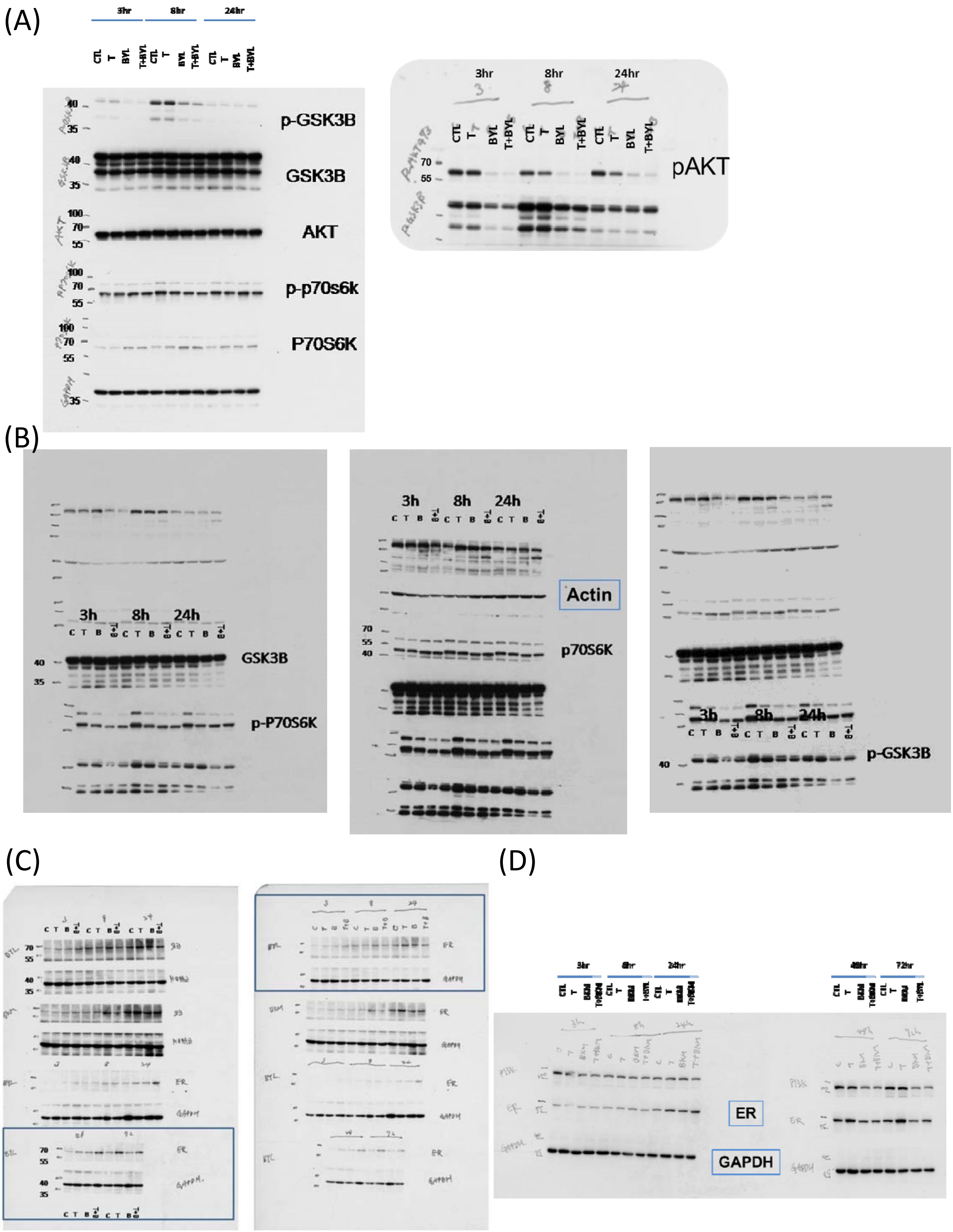
(A)
3hr
8hr
24hr
CTL
T
BYL
T+BYL
p-GSK3B
GSK3B
AKT
p-p70s6k
P70S6K
pAKT
(B)
3h
8h
24h
GSK3B
p-P70S6K
Actin
p70S6K
p-GSK3B
(C)
(D)
3hr
8hr
24hr
48hr
72hr
ER
GAPDH

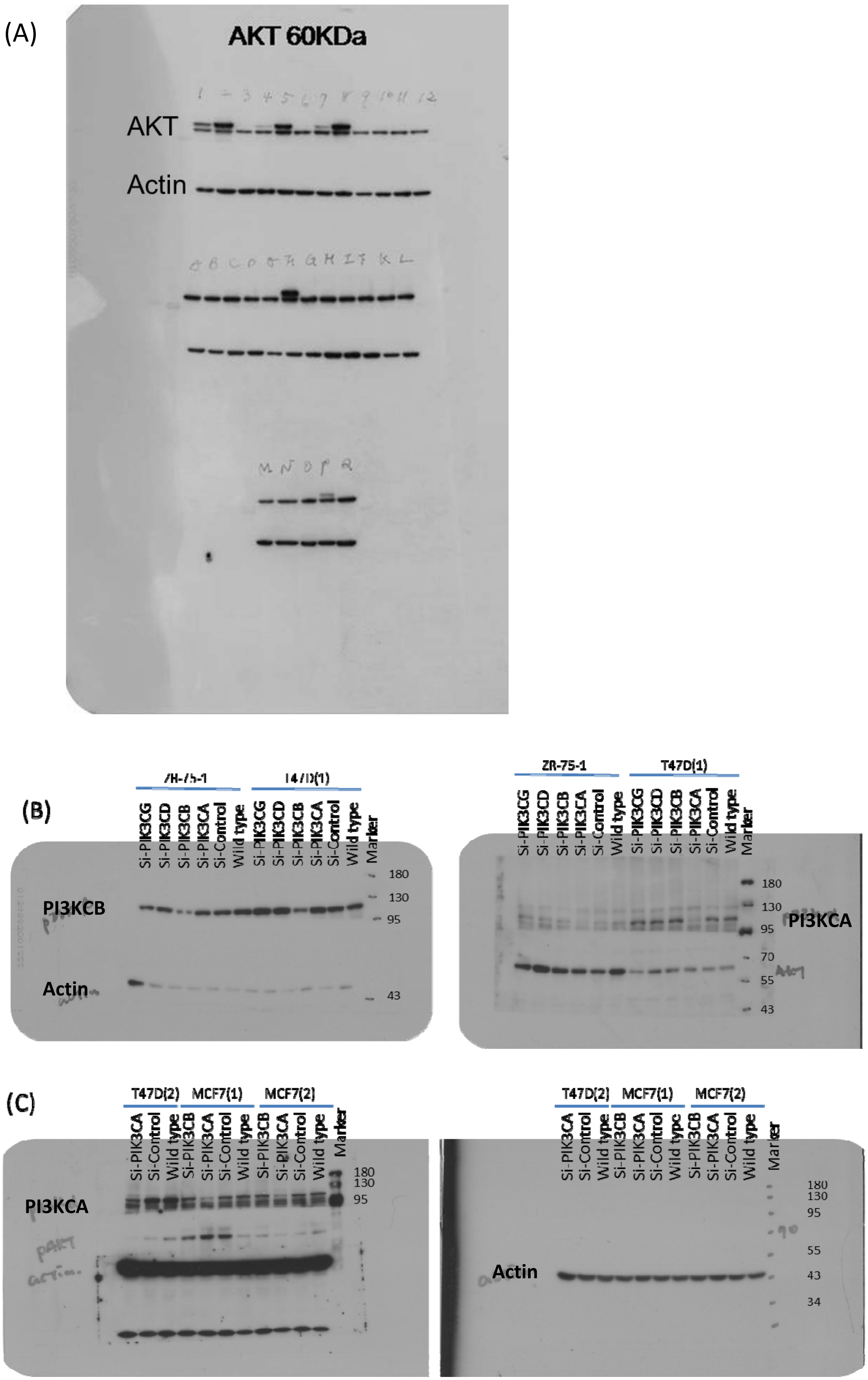

(A)
AKT 60KDa
AKT
Actin
(B)
ZR-75-1
T47D(1)
Si-PIK3CG
Si-PIK3CD
Si-PIK3CB
Si-PIK3CA
Si-Control
Wild type
Marker
PI3KCB
Actin
180
130
95
43
PI3KCA
70
55
(C)
T47D(2)
MCF7(1)
MCF7(2)
PI3KCA
Actin
34

| p-value | Control | T | BYL719 | BKM120 |
| --- | --- | --- | --- | --- |
| T | 0.002199 | | | |
| BYL719 | 0.102642 | | | |
| BKM120 | 0.01985 | | | |
| T+BYL719 | 0.000921 | 0.001334 | 0.000815 | |
| T+BKM120 | 0.000666 | 0.000134 | | 0.003286 |
